# *BmS6K* Regulates Growth and Survival Traits of Silkworm, *Bombyx mori*

**DOI:** 10.3390/biology15211953

**Published:** 2026-10-30

**Authors:** Linrong Wan, Bowen Yin, Rui Xi, Yuanyuan Wen, Lin Wang, Mengyan Liu, Xiaoling Tong, Dingpei Long, Fangyin Dai

**Affiliations:** 1 State Key Laboratory of Resource Insects, Key Laboratory of Sericultural Biology and Genetic Breeding, Ministry of Agriculture and Rural Affairs, College of Sericulture, Textile and Biomass Sciences, Southwest University, Chongqing 400715, China; 2 Sericultural Research institute, Sichuan Academy of Agricultural Sciences (SAAS), Nanchong 637000, China

**Keywords:** *Bombyx mori*, *BmS6K*, growth regulation, CRISPR/Cas9, transgenesis, mTOR signaling

## Abstract

Ribosomal protein S6 kinase (S6K) is a conserved effector of the mTOR signaling pathway that regulates cell growth and metabolism. The organism-level function of S6K in the silkworm *Bombyx mori* has not yet been examined by direct genetic manipulation. Here, we combined CRISPR/Cas9-mediated knockout, *piggyBac*-based transgenic re-expression and overexpression, and multi-tissue RNA-seq with weighted gene co-expression network analysis (WGCNA) to characterize the role of *BmS6K* in vivo. *BmS6K*-null mutants were viable but showed 26.5% and 28.6% reductions in pupal weight in males and females, respectively. Furthermore, mutant survival rates were 44.2% (males) and 28.5% (females) lower than those of controls, with mortality concentrated at the late fifth larval stage. Targeted transgenic re-expression rescued these phenotypic defects. Conversely, overexpressing *BmS6K* in a wild-type background increased the larval growth rate by 16.9% and significantly raised both whole cocoon weight and pupal weights. WGCNA of the three tissue types identified a core co-expression module (MEred) that was significantly enriched for genes involved in proton-transporting V-type ATPase activity and vacuolar complex assembly, mTOR signaling, and oxidative phosphorylation coupled to cellular redox homeostasis, including BmV1-ATPase, BmGabaT, and BmNadDac, whose orthologs represent core components or mediate critical cross-talk in the TOR-S6K pathway, a function conserved in *Drosophila* and mammals. Since the loss and gain of *BmS6K* produced opposite effects on growth and viability, it can be regarded as a candidate target for increasing cocoon yield in sericulture, as well as a target for RNAi-based control of lepidopteran pests through the suppression of its orthologs.

## Simple Summary

Silkworms are among the most economically important domesticated insects. Understanding the genetic mechanisms that regulate larval growth and survival may provide new opportunities for improving silk production. Ribosomal protein S6 kinase (S6K) is a highly conserved regulator of cell growth and metabolism, but its organism-level functions in the silkworm have not been directly established. Here, we combined CRISPR/Cas9-mediated knockout, transgenic rescue, and overexpression to evaluate the in vivo functions of *BmS6K*. Loss of *BmS6K* reduced pupal weight and survival, whereas transgenic rescue restored the major mutant phenotypes. *BmS6K* overexpression in a wild-type background accelerated late-larval growth and increased whole cocoon weight and pupal weights, supporting a positive role for *BmS6K* in growth-related traits. Multi-tissue transcriptomic analyses identified additional tissue-dependent transcriptional responses and candidate *BmS6K*-associated genes and pathways. These findings establish *BmS6K* as an important regulator of silkworm growth and provide a genetic basis for evaluating its potential in sericultural improvement.

## 1. Introduction

The S6 kinase (S6K) gene family, typified by ribosomal protein S6 kinase 1, serves as a core effector of cellular signaling networks in eukaryotes [1,2]. As a key downstream target of the mTORC1 pathway, the central regulator of nutrient sensing and energy homeostasis [3,4], S6K transduces nutritional and metabolic signals to drive protein synthesis, thereby regulating the core processes of cell growth, proliferation, and metabolic adaptation [5,6]. This growth-regulatory role has been highly conserved throughout eukaryotic evolution [1,2,7,8,9,10].

In insects, S6K has retained its conserved growth-modulatory functions while gaining lineage-specific functional features. In *Drosophila*, downregulation of S6k reduces body size, and its depletion significantly extends the adult lifespan, establishing a robust genetic link between S6K and the control of aging [11]. Beyond growth and longevity, S6K is essential for invertebrate reproductive physiology [12]. In the crustacean *Macrobrachium nipponense*, suppression of S6K expression disrupts ovarian development and impairs oocyte maturation, mirroring corresponding phenotypes observed in mammals [13]. Transcriptomic profiling under starvation further positions S6K as a core downstream effector of mTORC1 and an integration node for ERK7 pathway signaling that mediates adaptive responses to nutritional stress in insects [14,15]. However, in vivo genetic evidence for S6K function remains lacking in economically important *Lepidoptera*.

The domestic silkworm, *Bombyx mori*, is both an economically important insect globally and a well-established lepidopteran model organism [16,17]. As a fully domesticated species central to the global textile industry, the genetic traits of silkworms have undergone long-term artificial selection to improve silk yield [18,19]. Silkworms exhibit rapid larval growth and an exceptionally high capacity for protein synthesis, with well-defined economic traits tied to silk production. The species also has a mature CRISPR genome editing and transgenesis platform, alongside rich multi-omic resources [20]. These features make it a highly suitable system for investigating insect growth-regulatory mechanisms and translating research findings into trait improvement.

At present, studies involving the functional characterization of *BmS6K* in *B. mori* remain limited. Prior studies have only inferred its involvement in BmNPV infection from expression profiling data [21,22], whereas its direct effects on silkworm growth and developmental progression have not been systematically examined, and definitive in vivo genetic evidence via targeted gene manipulation is absent [23]. Two fundamental questions remain to be addressed. First, does *BmS6K* causally regulate organismal growth and survival in *B. mori*? Second, what tissue-specific transcriptional alterations are elicited by *BmS6K* deficiency? To resolve these questions, we employed an integrated strategy combining *BmS6K* knockout, transgenic overexpression, genetic rescue, and multi-tissue transcriptomic profiling to systematically dissect the growth-regulatory function of *BmS6K*. Our findings will inform future genetic improvement of growth-related traits in silkworms and may have broader implications for the genetic improvement of other economically important insects and the development of targeted pest management strategies.

## 2. Materials and Methods

### 2.1. Silkworm Strains and Genome Data

The silkworm strain Dazao was reared under standard laboratory conditions at 25 ± 1.0 °C with a 12:12 h light/dark photoperiod and 50–70% relative humidity. Larvae were fed fresh mulberry leaves ad libitum to ensure uniform growth and development. All downstream genomic analyses were aligned to the *B. mori* reference genome assembly, and the corresponding gene annotation set from the same build was retrieved from SilkBase (https://silkbase.ab.a.u-tokyo.ac.jp, SilkBase-assembly-Nov-2016, accessed on 1 October 2025).

### 2.2. Spatiotemporal Expression Pattern and Evolutionary Analysis

Expression profiles for the ribosomal protein S6 kinase gene were extracted from the EXP832 resource [17]. Lepidopteran orthologs of *BmS6K* (KWMTBOMO05749/BGIBMGA006807/LOC100174852) were retrieved from the OrthoDB database [24], and the *Drosophila* S6K protein sequence (CG_10539) was included for comparative analysis. For genes with multiple transcript isoforms, the protein sequence encoded by the longest transcript was selected for downstream analyses. Multiple protein sequence alignment was performed using the MUSCLE algorithm integrated into MEGA11 software [25]. A phylogenetic tree was constructed using the maximum likelihood (ML) method under the LG + G + I amino acid substitution model, with 1000 bootstrap replicates used to assess the reliability of tree topology. The resulting phylogenetic tree was visualized and annotated using the ggtree package in R (v4.0.5) [26]. The functional domains of *BmS6K* were predicted using the SMART tool (v10.0.0) [27], and collinearity analysis of the *BmS6K* genomic locus was conducted with MCScanX (v1.0.0) [28]. All alignment segments were visualized using the aplot R package(v0.2.0) [29].

### 2.3. RNA Extraction, cDNA Synthesis, and qRT-PCR

Total RNA was extracted from fifth-instar silkworm larvae (days 1–6) using the Fast RNAex Kit (BGMG, Chengdu, China) and mulberry leaf residues in the midgut were removed before RNA extraction. For each biological replicate, RNA was pooled from five male and five female fifth-instar larvae (mixed sexes), with three biological replicates per sample. Approximately 1 µg of total RNA was used as input for cDNA synthesis. Contaminating genomic DNA was removed using the gDNA Eraser included in the PrimeScript™ RT reagent Kit (TAKARA, Kyoto, Japan), and reverse transcription to complementary DNA (cDNA) was then performed with the 5× PrimeScript mix.

Quantitative real-time polymerase chain reaction (qPCR) was performed on a Rotor-Gene instrument (QIAGEN, Hilden, Germany) using a SYBR qPCR kit (Accurate Biology/AG, Changsha, China), following the manufacturer’s protocols, with an initial denaturation at 95 °C for 30 s followed by 40 cycles of 95 °C for 5 s and 60 °C for 30 s. Three biological replicates were prepared for each sample and each was assayed in three technical replicates to ensure data reliability. The relative expression levels of target genes were calculated using the 2^−ΔΔCt^ method, with Sw22934 as the endogenous reference gene for normalization. Sw22934 encodes the silkworm eukaryotic translation initiation factor 4A (eIF4A; accessions: KWMTBOMO02081, BGIBMGA003186, LOC692781) and has been widely used for qRT-PCR normalization in silkworm studies; its stability was validated across the developmental stages and tissues examined in this study. Primer efficiency was determined and incorporated into the calculation of transcript abundance, and melt curve analysis (65–95 °C, 0.5 °C per 5 s) was performed to confirm amplicon specificity. All the primer sequences are listed in Table S1.

### 2.4. CRISPR/Cas9-Mediated Knockout

To generate the *BmS6K* knockout silkworm line, we designed two single-guide RNAs (sgRNAs) targeting the *BmS6K* coding region using CRISPR-direct. The two sgRNAs, *BmS6K*_grna1 and *BmS6K*_grna2 (Table S1), were synthesized by Tsingke Biotechnology (Chongqing, China). Transcription templates for sgRNAs were amplified by PCR with the universal primer CRISPR_sgR (Table S1) using PrimeSTAR GXL DNA polymerase (Takara), with an initial denaturation at 98 °C for 2 min, followed by 35 cycles of 98 °C for 10 s, 55 °C for 15 s, and 68 °C for 15 s, and a final extension at 68 °C for 5 min. The amplified products were transcribed in vitro using the TranscriptAid T7 Kit (Thermo Fisher Scientific, Waltham, MA, USA). The purified sgRNAs (0.5 µL each) were incubated with 2.5 µg (0.5 µL) of Cas9 Protein (Invitrogen, Thermo Fisher Scientific) at 37 °C for 30 min to form ribonucleoprotein complexes, at a Cas9:sgRNA volume ratio of 1:2, which were then microinjected into freshly de-diapaused silkworm eggs within 2 h of electrical activation [30,31].

The strain used in this study, Dazao, is an egg-diapause strain, and in our laboratory diapause is routinely terminated by electrical activation of the eggs as described previously [30,31]. An online tool based on R Shiny (Sangerseq_shiny: https://wanlinrong.shinyapps.io/sangerseq_shiny, accessed on 1 March 2026) was used for high-throughput genotyping after microinjection by processing *.ab1 sequencing files.

After microinjection, the G0 larvae were reared individually under standard laboratory conditions. Upon adult eclosion, genomic DNA was extracted from the wings of the moths, and genotyping was performed via PCR using the primer pair *BmS6K*_checking_F/R (Table S1). G0 individuals carrying indel alleles verified by superimposed sequencing peaks were outcrossed with wild-type moths to generate G1 progeny. G1 individuals with identical frameshift indel alleles were then intercrossed to produce homozygous G2 mutants. Sibling mating of these G2 mutants was subsequently performed to establish a stable *BmS6K*^−/−^ homozygous knockout line for downstream phenotypic and molecular analyses.

### 2.5. PiggyBac Transposon-Mediated Overexpression

The full-length coding sequence (CDS) of *BmS6K* was commercially synthesized by Tsingke Biotechnology Co., Ltd. (Chongqing, China). The CDS was amplified from the synthesized vector by PCR using PrimeSTAR GXL DNA polymerase (Takara) with primers that introduced BamHI and NotI restriction sites and homology arms at its 5′ and 3′ termini (Table S1; lowercase letters indicate homology arms) under the same conditions described for sgRNA template amplification. The PSL1180 intermediate vector was linearized by double digestion with BamHI and NotI, and the amplified CDS was inserted by homologous recombination. The IE1-*BmS6K*-SV40 expression cassette was then amplified from the verified recombinant PSL1180 plasmid [32], appended with 15-bp homologous arms at both ends, and integrated into the *piggyBac* (pBac) transposon backbone via homologous recombination to generate the final overexpression vector pBac[IE1-*BmS6K*-SV40, 3 × P3-EGFP-SV40] (Figure S1). The recombinant pBac vector was mixed with the A3 helper plasmid [32] at a 1:1 volume ratio to a final concentration of approximately 500 ng/μL and microinjected into preblastoderm silkworm eggs within 2 h post-oviposition. The injected eggs were sealed and incubated at 25 ± 1.0 °C and 80% relative humidity (Figure S1).

*BmS6K* overexpression strains were initially established in a *BmS6K* heterozygous knockout background. Putative transformants were validated through three hierarchical assays. The first was preliminary screening by 3 × P3-driven EGFP fluorescence in the compound eyes, followed by genomic insertion site mapping using Wristwatch-PCR [33] (wwPCR, Table S2), and finally transcriptional overexpression confirmation via qRT-PCR. Multiple generations of self-crossing and genotypic segregation ultimately established two stable overexpression lines. The line with a homozygous *BmS6K* knockout background was used for the genetic rescue assay, while the line with the wild-type background was applied to phenotypic analysis of *BmS6K* overexpression (Figure S2).

### 2.6. Phenotypic Characterization

All phenotypic assays were performed using newly molted fifth-instar *B. mori* larvae maintained on a uniform ad libitum feeding regime. For the *BmS6K* knockout experiment, the wild-type Dazao strain and the *BmS6K* knockout strain were reared separately as the control and experimental groups, respectively. Each group comprised three biological replicates with 15 larvae per replicate. For the overexpression assay, the *BmS6K* overexpression strain and its corresponding control strain were co-reared within the same rearing containers to eliminate environmental confounding factors. Each container housed 15 control larvae and 15 overexpression larvae, across three biological replicates; the two strains were externalized and distinguished via fluorescence microscopy. Phenotypic measurements were recorded at 12-h or 24-h intervals from hour 0 to day 7 of the fifth instar. Whole cocoon weight, pupal weight, and cocoon shell weight were quantified 5 days after cocoon formation.

### 2.7. Sample Collection, Transcriptome Sequencing, and Data Preprocessing

Tissues critical for organismal growth, developmental progression, and environmental adaptability, including the head, midgut, and fat body, were harvested from wild-type (WT, None) and *BmS6K* knockout (KO, CRISPR-Cas9) silkworm larvae at day 3 of the fifth instar (5L3D). These three tissues were selected because the head represents the neuroendocrine regulation and sensory center, the midgut represents digestion, nutrient absorption, detoxification, and the immune barrier, and the fat body represents the metabolic center, energy storage, and basal biosynthetic metabolism; together, they cover the growth regulation, metabolic, and immune-related pathways in which *BmS6K* may act. Three independent biological replicates were included, resulting in a total of 18 transcriptome samples (two genotypes × three tissues × three biological replicates); each biological replicate was pooled from five male and five female larvae at this stage. Total RNA was isolated from each sample for RNA sequencing (RNA-seq) analysis, which was performed on the DNBSEQ-T7 platform at a sequencing service facility in Chengdu, China. The raw sequencing data have been deposited in NCBI under BioProject accession **PRJNA1526990**.

Downstream bioinformatics analysis of the sequencing data was performed as follows. The raw sequencing reads first underwent quality control preprocessing using fastp (v0.23.2) [34] during which adapter sequences and low-quality bases were trimmed and filtered. After preprocessing, the clean reads were mapped to the *B. mori* reference genome using HISAT2 (v2.2.1) [35] with default parameters. Transcriptome assembly and quantification of gene expression were performed using StringTie (v2.2.1) [36], generating raw read counts and transcripts per million (TPM) expression values. To evaluate the reproducibility of expression profiles across samples, clustering analysis was conducted based on the TPM expression matrix using the R packages ggtree [37] and tvBOT(v2.6.1) [38].

### 2.8. Identification of Differentially Expressed Genes and Functional Enrichment Analysis

Differential gene expression analysis was performed using the Bioconductor DESeq2 package (v1.50.2) [39], with raw read count data as input. Genes were classified as significantly differentially expressed genes (DEGs) if they met the dual thresholds of an absolute log2 fold change (|log_2_FC|) ≥ 2 and an adjusted *p*-value (padj) ≤ 0.05. Functional enrichment analysis of DEGs was carried out using the DAVID database [40,41], covering Gene Ontology (GO) terms across the three categories of biological process, molecular function, and cellular component, as well as Kyoto Encyclopedia of Genes and Genomes (KEGG) pathway annotations. Enrichment terms with a padj value ≤ 0.05 were considered statistically significant. All enrichment results were visualized using the ChiPlot online analytical platform (https://www.chiplot.online, accessed on 10 June 2026).

### 2.9. Weighted Gene Co-Expression Network Construction and Hub Gene Mining

Weighted gene co-expression network analysis (WGCNA) was performed using the WGCNAshiny module (v0.0.5.220808) integrated into TBtools (v2.4.6) [42,43], based on the RNA-seq count matrix. Prior to network construction, gene expression counts were normalized using the variance-stabilizing transformation (VST) method in DESeq2 [39]. To improve network resolution, genes with low expression variability, as determined by median absolute deviation (MAD), were removed, and the top 8000 most variable genes were retained for downstream analysis.

The topology of the network was evaluated by calculating the scale-free fit index (R^2^) and mean connectivity to select an optimal soft-thresholding power (β). Pairwise correlation coefficients were computed to generate a topological overlap matrix (TOM), which served as the basis for hierarchical clustering. Gene modules were identified using the dynamic tree-cutting algorithm. Module eigengenes were subsequently correlated with phenotypic traits to screen for biologically relevant modules. Hub genes were defined as those with both high intramodular connectivity (|KME| ≥ 0.9) and strong phenotypic association (|GS| ≥ 0.8).

Functional enrichment analysis was performed using the DAVID database [40], and gene name mapping was conducted via the SGID database [44] (http://sgid.popgenetics.net/, accessed on 6 July 2026). Protein–protein interaction networks were subsequently constructed and analyzed using the STRING database [45].

### 2.10. Statistical Analysis

Statistical analyses for all datasets were performed in R (v4.5.3) or Python (v3.11.17). All values are presented as mean ± standard error of the mean (SEM), derived from no fewer than three independent biological replicates. Unpaired two-tailed Student’s *t*-tests were used for intergroup comparisons unless otherwise noted.

## 3. Results

### 3.1. Expression Profile of BmS6K and Its Evolutionary Conservation Across Lepidoptera

**Figure 1:**
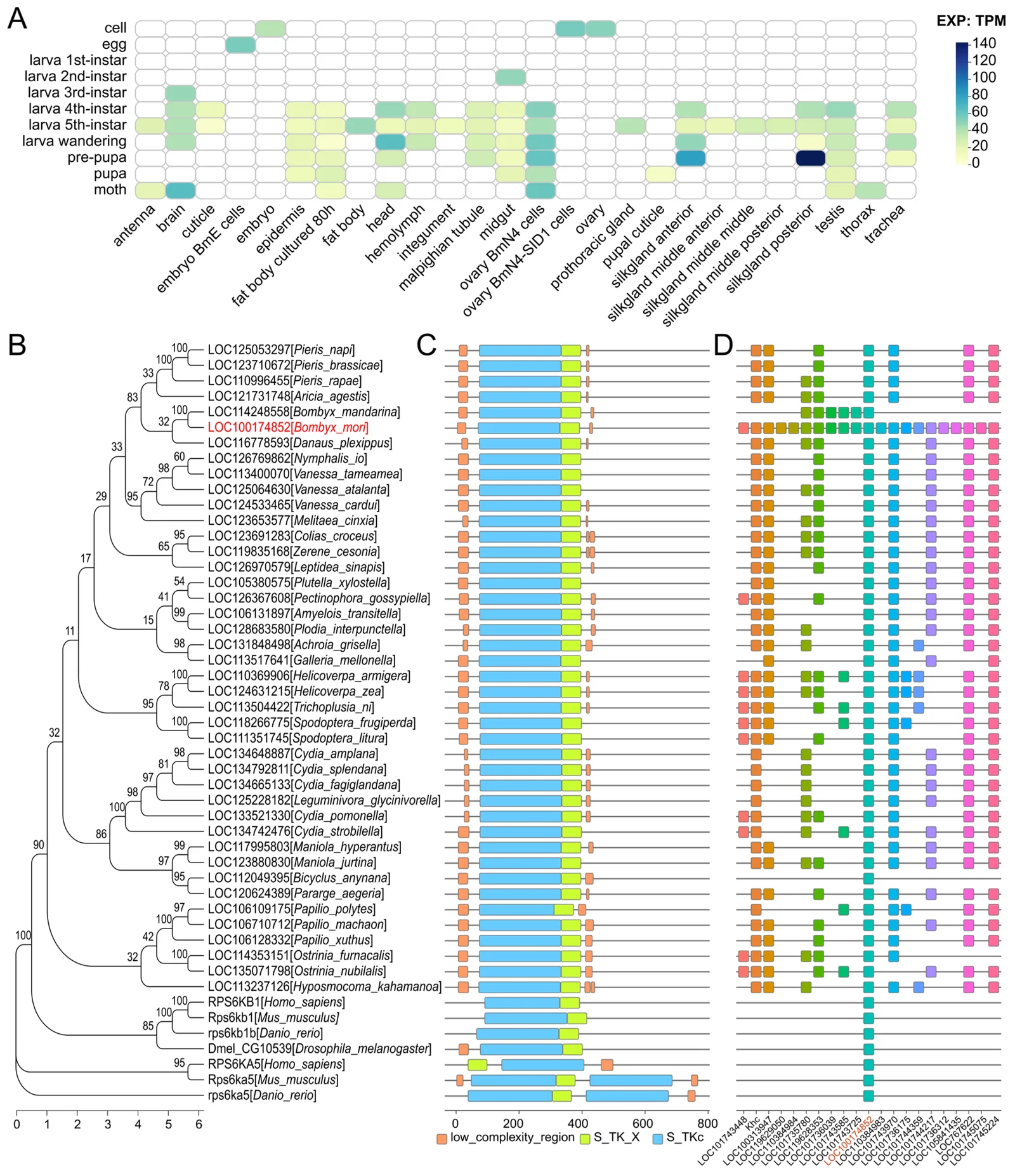
Spatiotemporal expression profile and evolutionary analysis of *BmS6K*. (**A**) Spatiotemporal expression heatmap of *BmS6K* in *B. mori*. Rows indicate developmental stages, and columns indicate tissues and cultured cell lines. The color scale represents TPM values of gene expression. (**B**) Phylogenetic tree of S6K homologous proteins from various species. (**C**) Domain organization of S6K proteins in representative species. The colored blocks denote functional domains and low-complexity regions. (**D**) Synteny analysis of the S6K genomic region across species. The colored boxes indicate a silkworm gene locus used to display synteny.

We first characterized the spatiotemporal expression profile of *BmS6K* across developmental stages and tissues based on the EXP832 transcriptome data source (Figure 1A). Transcript abundance peaked in the posterior silk gland at the prepupal stage. *BmS6K* was broadly expressed during larval development, with relatively high levels in the midgut, head, fat body, and silk gland from the fourth instar to the wandering stage. In contrast, overall expression was weak during the embryonic and pupal stages. Notably, besides the posterior silk gland at the prepupal stage, relatively high expression was also detected in the gonads (ovary and testis). However, fertility-related phenotypes were not significantly affected by *BmS6K* perturbation.

To investigate the evolutionary conservation of S6K, we conducted phylogenetic reconstruction, protein domain comparisons, and genomic synteny analyses across diverse species. Phylogenetic analysis of S6K homologous proteins demonstrated that *BmS6K* grouped closely with homologs from other lepidopteran species, forming a well-supported lepidopteran-specific clade (Figure 1B). Comparative analysis of the protein domain architecture showed that the core functional domains of S6K were uniformly preserved across all representative species, displaying a highly conserved structural arrangement (Figure 1C). A synteny analysis of the S6K genomic locus revealed conserved flanking gene organization across species (Figure 1D). The results indicated that S6K has been evolutionarily conserved at both the protein sequence and genomic structure levels in *Lepidoptera*.

### 3.2. CRISPR/Cas9-Mediated BmS6K Knockout and Identification

**Figure 2:**
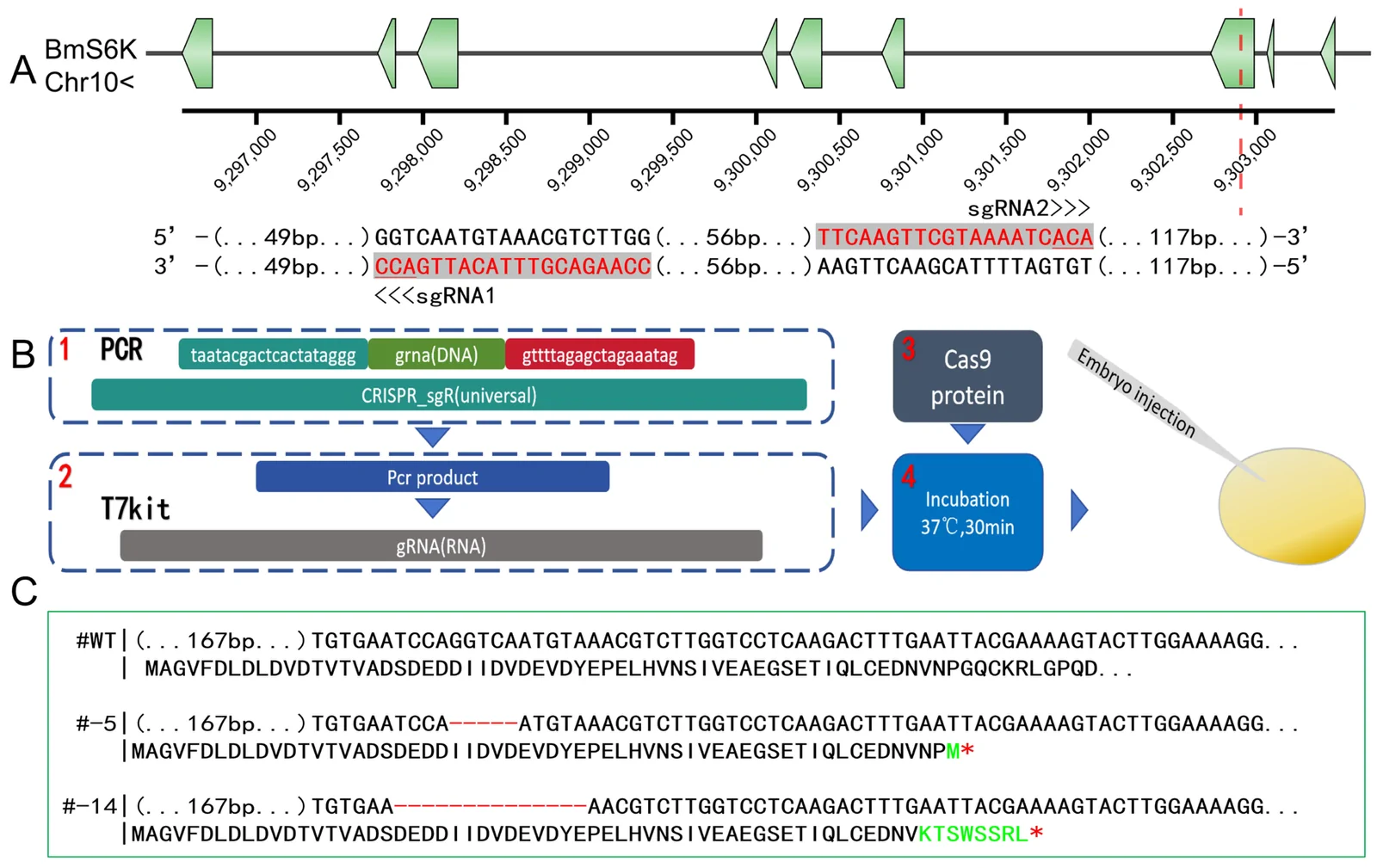
Generation and verification of *BmS6K* knockout mutants. (**A**) Genomic structure of the *BmS6K* locus on chromosome 10. The green boxes indicate exons, and the red dashed lines mark the sgRNA targeting sites. Sequences marked in red indicate the sgRNA target sites on both DNA strands. (**B**) Schematic overview of the CRISPR/Cas9-mediated gene knockout workflow. (**C**) Sequence confirmation of *BmS6K* mutant alleles. The (**upper**) panel shows the positions of the two sgRNA target sequences. The (**lower**) panel shows the alignment of genomic and deduced amino acid sequences from the wild type (WT) and two representative mutant lines. Gaps represent deleted nucleotides, green letters represent the newly encoded amino acids resulting from the frameshift, and asterisks indicate premature stop codons induced by frameshift mutations.

To explore the functional role of *BmS6K* in silkworm development, we generated *BmS6K* knockout mutants using the CRISPR/Cas9 genome editing system. *BmS6K* is a multi-exon locus located on chromosome 10, comprising nine exons, and two sgRNA target sites (*BmS6K*_grna1 and *BmS6K*_grna2, Table S1) were designed within the CDS region of the third exon to introduce frameshift mutations (Figure 2A). The knockout procedure was implemented by assembling the Cas9-sgRNA ribonucleoprotein complex and performing microinjection into early silkworm embryos (Figure 2B).

Two independent stable mutant lines (#-5 and #-14) were obtained by screening and homozygous breeding. Sanger sequencing confirmed that both alleles carried nucleotide deletions at the target region that resulted in frameshift mutations in the open reading frame. These mutations introduced premature termination codons, leading to the production of truncated and functionally inactivated *BmS6K* proteins (Figure 2C). Line #-5 was selected for subsequent functional investigation.

### 3.3. Loss of BmS6K Impairs Silkworm Growth and Survival

**Figure 3:**
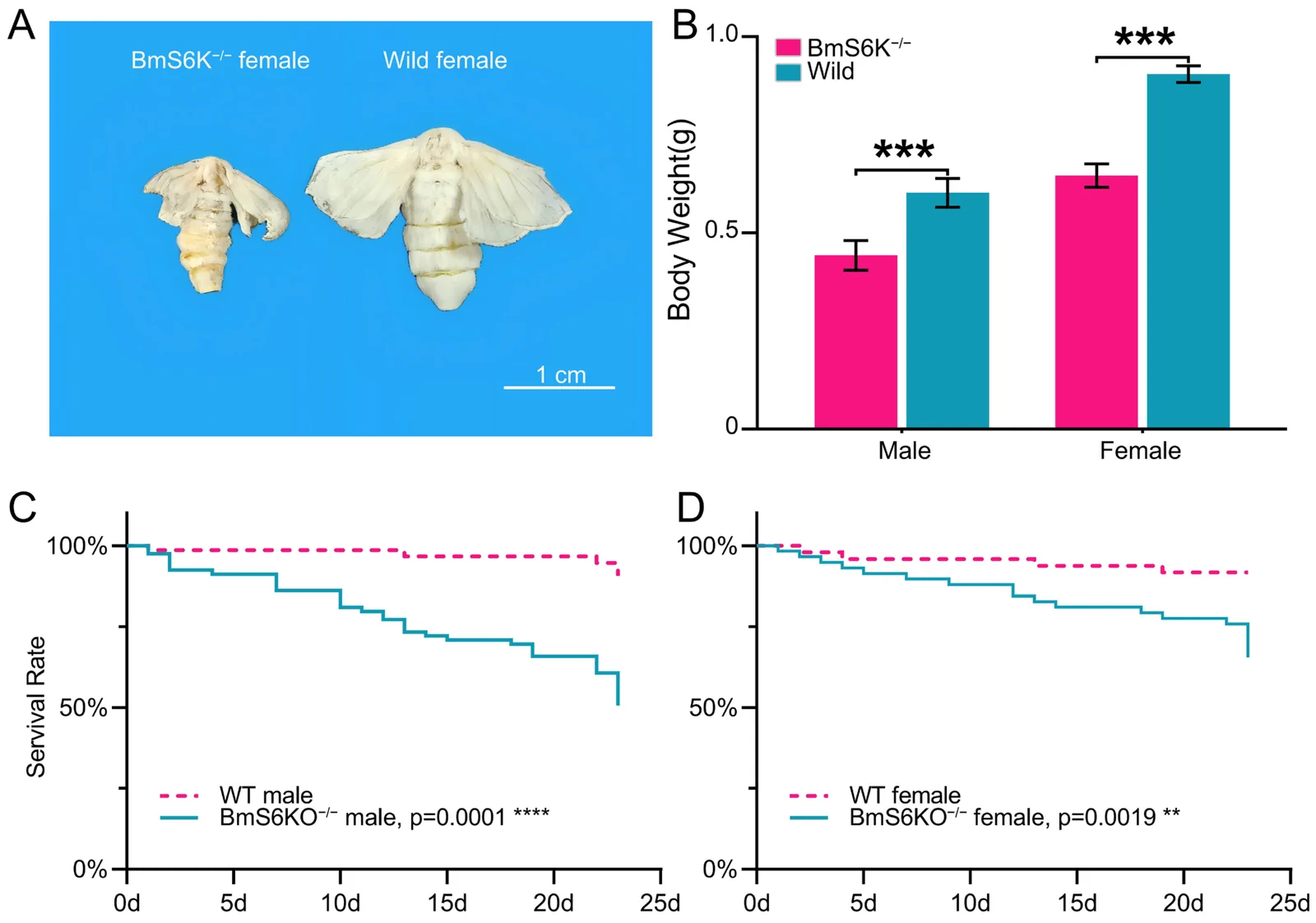
Effects of *BmS6K* knockout on adult morphology, pupal weight and survival capacity. (**A**) Phenotypic comparison of female adults between the wild-type (WT) and *BmS6K* homozygous knockout (*BmS6K*^−/−^) strains. Scale bar = 1 cm. (**B**) Comparison of pupal weights of male and female individuals. Data are presented as mean ± standard error of the mean (SEM). *** denotes a highly significant difference (*p* < 0.001). (**C**,**D**) Kaplan–Meier survival curves of male (**C**) and female (**D**) silkworms over a 25-day observation period. ** indicates a significant difference at *p* < 0.01, and **** indicates an extremely significant difference at *p* < 0.0001.

We next examined the consequences of *BmS6K* knockout on organismal growth and survival. Morphological observation showed that adult body size was visibly diminished in homozygous *BmS6K* knockout females compared with wild-type controls (Figure 3A). Similarly, measurement of pupal weight revealed a substantial growth defect in knockout individuals. Male and female knockouts retained only 73.5% and 71.4% of the wild-type pupal weight, representing reductions of approximately 26.5% and 28.6%, respectively. Both genotypic differences were highly significant (Figure 3B).

Kaplan–Meier survival analysis over a 25-day period demonstrated that the disruption of *BmS6K* markedly reduced survival in both sexes. By the end of the observation period, the final survival rate was 50.6% in *BmS6K* knockout males versus 90.70% in wild-type males, and 65.5% in knockout females versus 91.67% in wild-type females. Log-rank tests confirmed significant divergence in survival curves between the two genotypes for both males and females (Figure 3C,D). These findings indicate that functional loss of *BmS6K* severely impairs both growth performance and survival in silkworms.

### 3.4. Transcriptome Profiling Unravels Genes and Pathways Regulated by BmS6K

**Figure 4:**
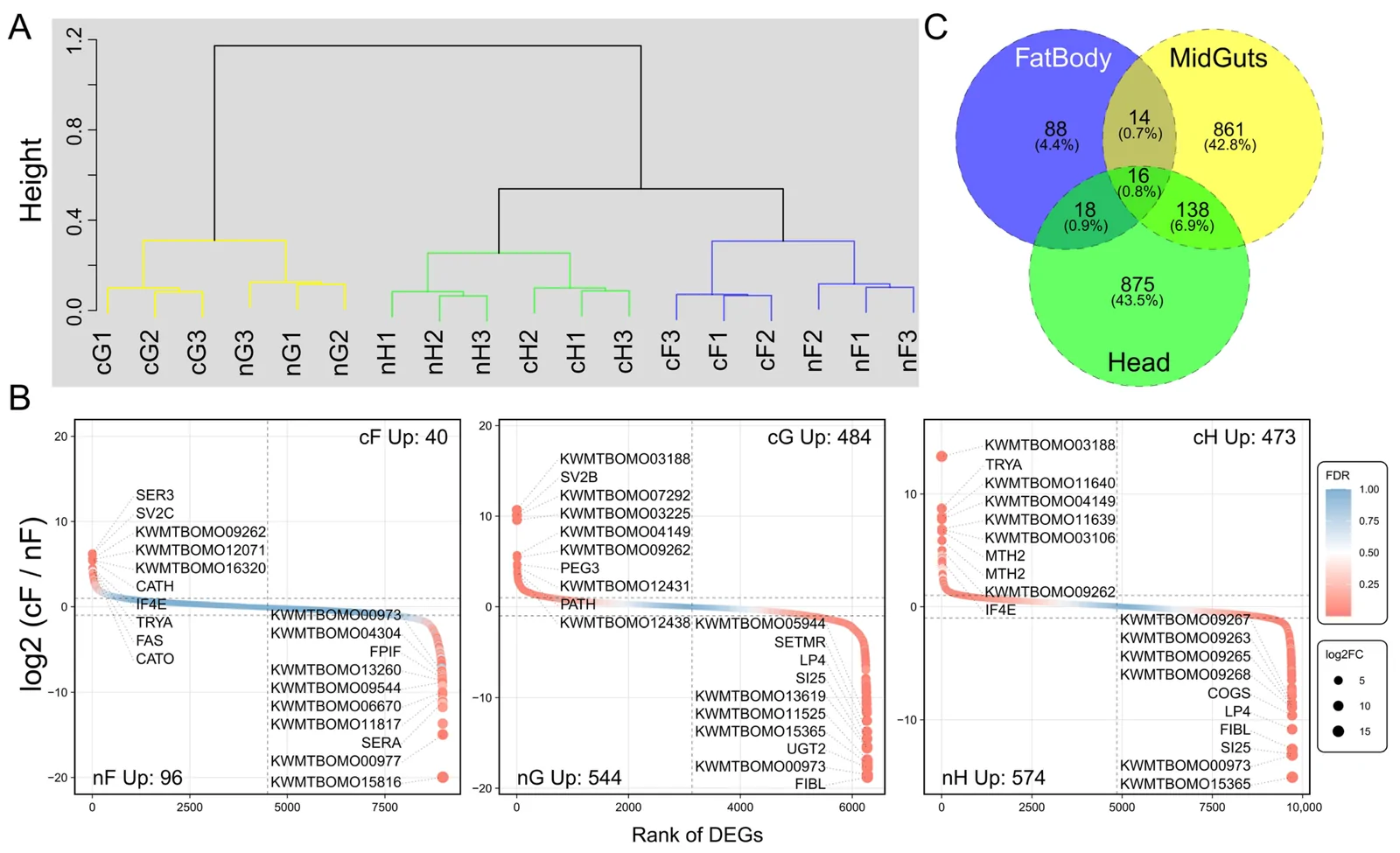
Sample clustering and differential gene expression analysis. (**A**) Hierarchical clustering dendrogram generated from genome-wide gene expression profiles of all samples. (**B**) Rank-ordered plots of differentially expressed genes for three pairwise comparisons. (**C**) Venn diagram depicting overlapping and tissue-specific differentially expressed genes.

To characterize the genome-wide transcriptional alterations associated with *BmS6K* across distinct tissues, we performed transcriptome sequencing on three representative tissues: fat body, midgut, and head. Unsupervised hierarchical clustering based on global gene expression profiles showed tight clustering of biological replicates within the same tissue and treatment group, confirming the reproducibility and reliability of the transcriptome dataset. Within each tissue, control and knockout samples were separated into distinct subclusters, indicating that *BmS6K* knockout was associated with tissue-specific transcriptomic changes (Figure 4A).

Pairwise differential expression analysis identified 136 differentially expressed genes (DEGs) in the fat body. Comparisons with the wild-type controls identified 40 upregulated genes and 96 downregulated genes in the knockout group. A total of 1028 DEGs were detected in the midgut of knockout larvae, with 484 upregulated and 544 downregulated. The head showed the most robust transcriptional response, yielding 1047 DEGs in total, with 473 upregulated and 574 downregulated in knockout samples (Figure 4B,C). Across all three tissues, the downregulated DEGs consistently outnumbered the upregulated DEGs in the knockout group, consistent with a predominant positive association between *BmS6K* and the expression of core transcriptional programs. The top 10 DEGs with the largest absolute fold changes were annotated for each comparison, and functionally characterized genes exhibited tissue-specific expression patterns closely related to the physiological functions of each tissue (Figure 4B).

As the core metabolic and energy-storage tissue in insects, the fat body displayed widespread downregulation of key metabolic genes following *BmS6K* knockout, including FAS for de novo lipogenesis, SER3 for serine biosynthesis, and CATH for proteolytic turnover, along with TRYA, IF4E, and CATO. This pattern indicates that *BmS6K* loss is associated with broad alterations in basal anabolism, protein processing, and translational efficiency in this tissue (Figure 4B). In the midgut, which is specialized for digestion, xenobiotic detoxification, and mucosal immunity, *BmS6K* knockout downregulated SV2B, PEG3, FIBL, and S25, while upregulating genes involved in detoxification and epigenetic regulation, including UGT2 and SETMR. These changes in expression reflect candidate-associated pathways related to stress response and translational machinery in the midgut (Figure 4B).

In the head, *BmS6K* knockout suppressed the expression of IF4E, TRYA, MTH2, S25, and FIBL, and induced the expression of COGS and LP4 (Figure 4B). Notably, S25 and FIBL were consistently downregulated following *BmS6K* knockout in both the midgut and head, representing genes with conserved *BmS6K*-responsive expression patterns across tissues. Other target genes showed tissue-specific regulatory patterns.

### 3.5. WGCNA Identifies Gene Co-Expression Networks Associated with BmS6K

**Figure 5:**
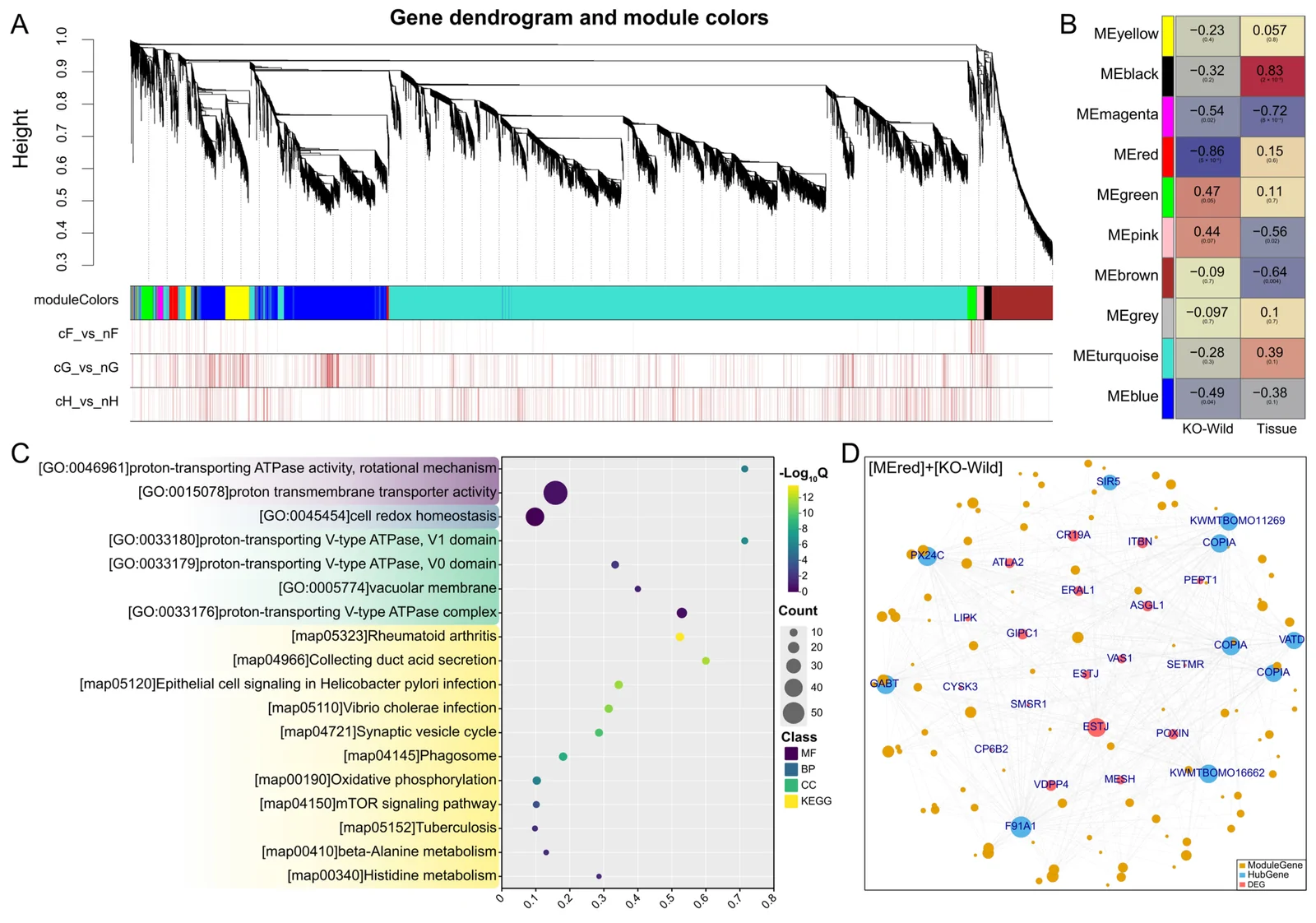
Weighted gene co-expression network analysis of the *BmS6K*-regulated transcriptome. (**A**) Gene dendrogram with module color annotation. The three lower tracks show the distribution of differentially expressed genes from three pairwise comparisons. (**B**) Heatmap of module-trait relationships. The rows represent color-named co-expression modules, and the columns correspond to two experimental traits. Each cell shows the Pearson correlation coefficient with its *p* value in parentheses. (**C**) Functional enrichment bubble plot. Dot size indicates the enriched gene count, and dot color represents the −log_10_-transformed Q value, covering GO categories and KEGG pathways. (**D**) Co-expression network of the MEred module. Node size scales with intramodular connectivity. The gray lines denote significant co-expression interactions, and representative genes are labeled with gene symbols.

We performed a WGCNA to systematically dissect the global gene co-expression networks governed by *BmS6K*. Based on genome-wide expression similarity (Figure S3), all genes were subjected to hierarchical clustering and partitioned into 10 independent co-expression modules (Figure 5A). Mapping of differentially expressed genes from the three tissue-wise comparisons below the module annotation strip revealed that *BmS6K*-responsive genes were not randomly distributed, but preferentially clustered in specific modules, indicating the existence of coordinated transcriptional programs in response to perturbation of *BmS6K*.

We then calculated the correlations between each module eigengene and the two experimental traits of *BmS6K* knockout status and tissue type to screen for trait-associated key modules (Figure 5B). The MEred module showed the strongest negative correlation with *BmS6K* knockout, with the highest statistical significance. This result suggested that genes in this module were coordinately suppressed after the depletion of *BmS6K*, and may form the core transcriptional network directly downstream of *BmS6K*. There was no significant correlation between MEred and tissue type, implying that this regulatory module acts as a universal effector of *BmS6K* across different tissues.

Functional analysis of MEred module genes showed significant enrichment in biological processes and pathways, including proton-transporting ATPase activity, V-type ATPase complex assembly, transmembrane transporter activity, oxidative phosphorylation, and lysosomal function (Figure 5C). These processes are tightly linked to cellular energy metabolism, substance transport, and anabolic homeostasis. We further visualized the intramodular co-expression network and identified genes with high connectivity as hub genes (Figure 5D). These hub genes, together with the differentially expressed genes highlighted in the network, represented high-priority candidates for subsequent mechanistic dissection of the *BmS6K* regulatory pathway (Table S4).

### 3.6. PiggyBac Transposon-Mediated BmS6K Overexpression and Molecular Validation

**Figure 6:**
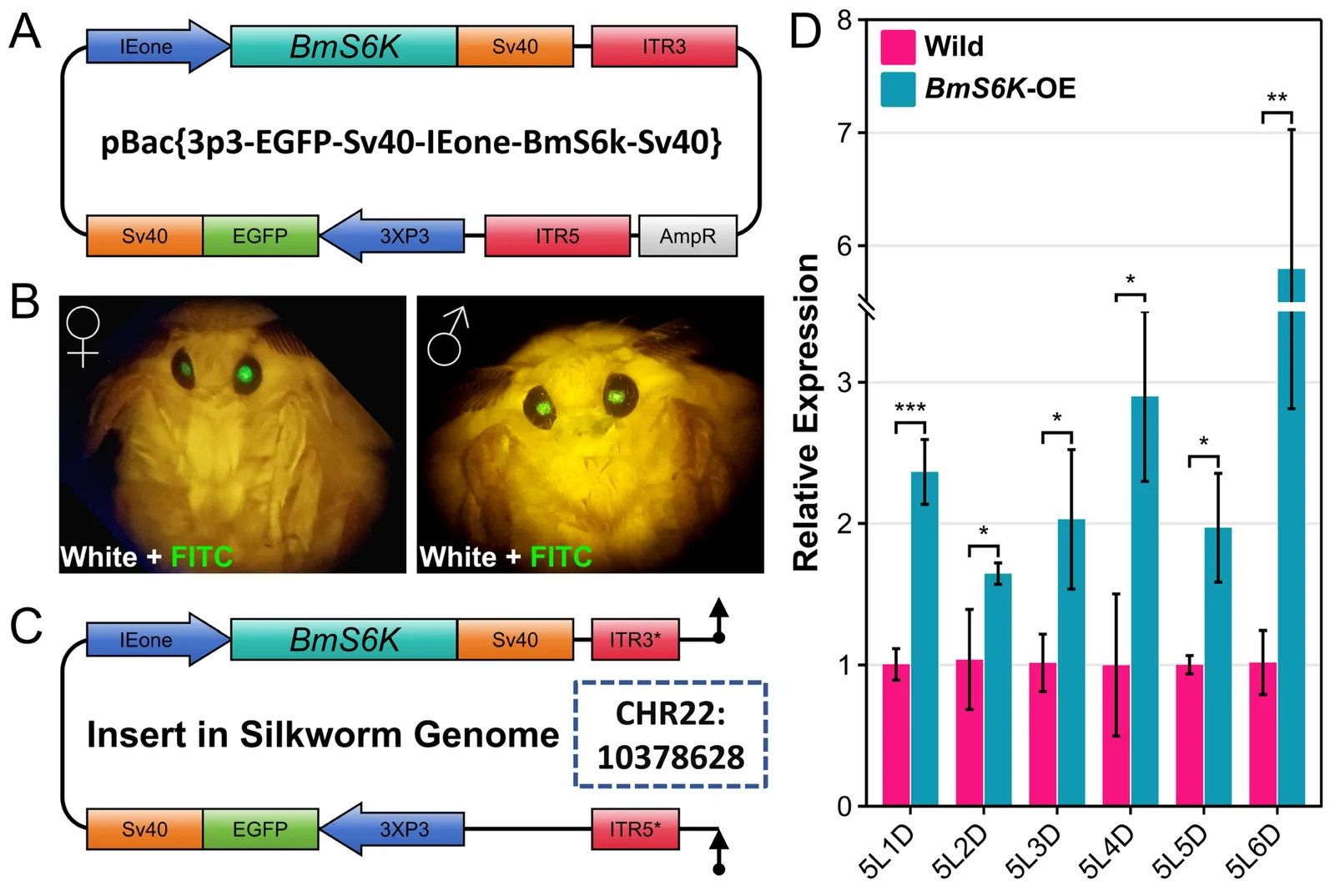
Construction and identification of the *BmS6K* over-expression strain. (**A**) Schematic diagram of the *piggyBac*-based transgenic vector ***piggyBac* [3×P3-EGFP-SV40-IE1-*BmS6K*-SV40]**. (**B**) EGFP fluorescence phenotype in the compound eyes of female and male transgenic moths. (**C**) Detection results of the transgene insertion site. (**D**) Quantitative real-time PCR assay of *BmS6K* expression in fifth-instar larvae of the S6KOE and control strains. * *p* < 0.05; ** *p* < 0.01; *** *p* < 0.001.

To generate *BmS6K* overexpression strains, G0 generation individuals obtained from microinjection of the specific plasmid were mated with wild-type silkworms (Figure 6A). G1 larvae were screened under a fluorescence microscope, and individuals with eye-specific EGFP fluorescence were selected as positive transformants (Figure 6B). After the G1 adults had matured, male and female individuals with fluorescence markers were selected for sibling mating. The fluorescence marker became stably inherited after several generations of selfing, and the transgene insertion site was identified via wwPCR (Table S2). The insertion site was located at position 10378628 on chromosome 22 (Figure 6C).

To confirm that *BmS6K* expression was indeed elevated in the transgenic strain, we extracted total RNA from whole larvae on days 1 to 6 of the fifth instar and performed qRT-PCR. Mulberry leaf residues in the midgut were removed before RNA extraction. The results showed that the *BmS6K* expression levels in the overexpression strain were consistently higher than those in the control group from day 1 to day 6 of the fifth instar, with statistically significant differences at multiple time points (*p* < 0.05; Figure 6D). The results confirmed the successful construction of the *BmS6K* overexpression strain.

### 3.7. Transgenic Re-Expression Rescues Growth and Survival Defects in BmS6K Knockout Mutants

**Figure 7:**
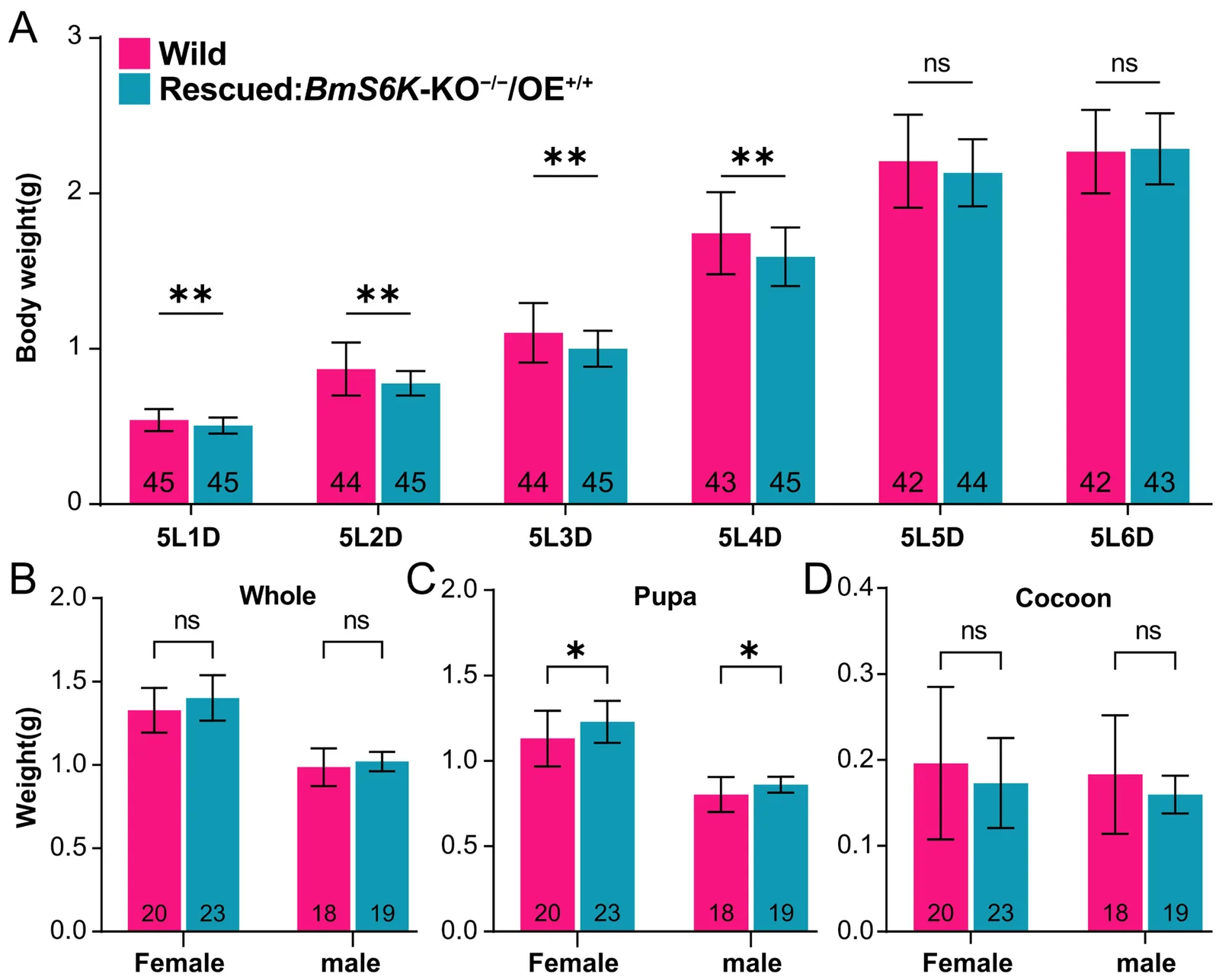
Phenotypic characterization of larval growth and cocoon–pupal traits in the S6K knockout-rescued silkworm line. (**A**) Dynamic body weight of fifth-instar larvae from day 1 to day 6 (5L1D–5L6D) in the S6K-rescued line (S6K-KO^−/−^/S6K-OE^+/+^) and the control. (**B**–**D**) Comparisons of whole-body weight (**B**), pupal weight (**C**), and cocoon weight (**D**) between the rescued line and CK, stratified by sex. Data are presented as mean ± SEM, with individual biological replicates shown as scattered dots. Sample sizes are labeled below each column. ** *p* < 0.01 and * *p* < 0.05; ns, not statistically significant.

To confirm that the delayed growth and decreased survival observed in *BmS6K* knockout mutants were directly caused by the loss of *BmS6K* function, we generated a genetic rescue line by reintroducing the full-length coding sequence into the *BmS6K*^−/−^ background via *piggyBac*-mediated transgenesis. Phenotypic verification showed that re-expression of *BmS6K* effectively rescued both the growth and survival defects induced by the gene knockout. Larvae of the rescue line (S6K-KO^−/−^/S6K-OE^)^ exhibited significantly lower body weight than wild-type Dazao controls (CK) from day 1 to day 4 of the fifth instar (5L1D to 5L4D, *p* < 0.01; Figure 7A). However, the rescue line displayed an accelerated growth rate as development proceeded, and the weight gap relative to the control gradually narrowed. By days 5 and 6 of the fifth instar (5L5D and 5L6D), the larval body weight of the rescue line was comparable to that of wild-type controls, with no statistically significant difference. We quantified both cocoon and pupal traits (Figure 7B–D). For both females and males, total cocoon weight and cocoon shell weight showed no significant difference between the rescue line and wild-type controls (ns). In contrast, pupal weight was significantly higher in the rescue line than in wild-type controls in both sexes (*p* < 0.05), indicating that *BmS6K* re-expression not only restored normal growth but also had an additional promoting effect on the accumulation of pupal biomass.

### 3.8. BmS6K Overexpression in a Wild-Type Background Enhances Larval Growth and Cocoon Traits

**Figure 8:**
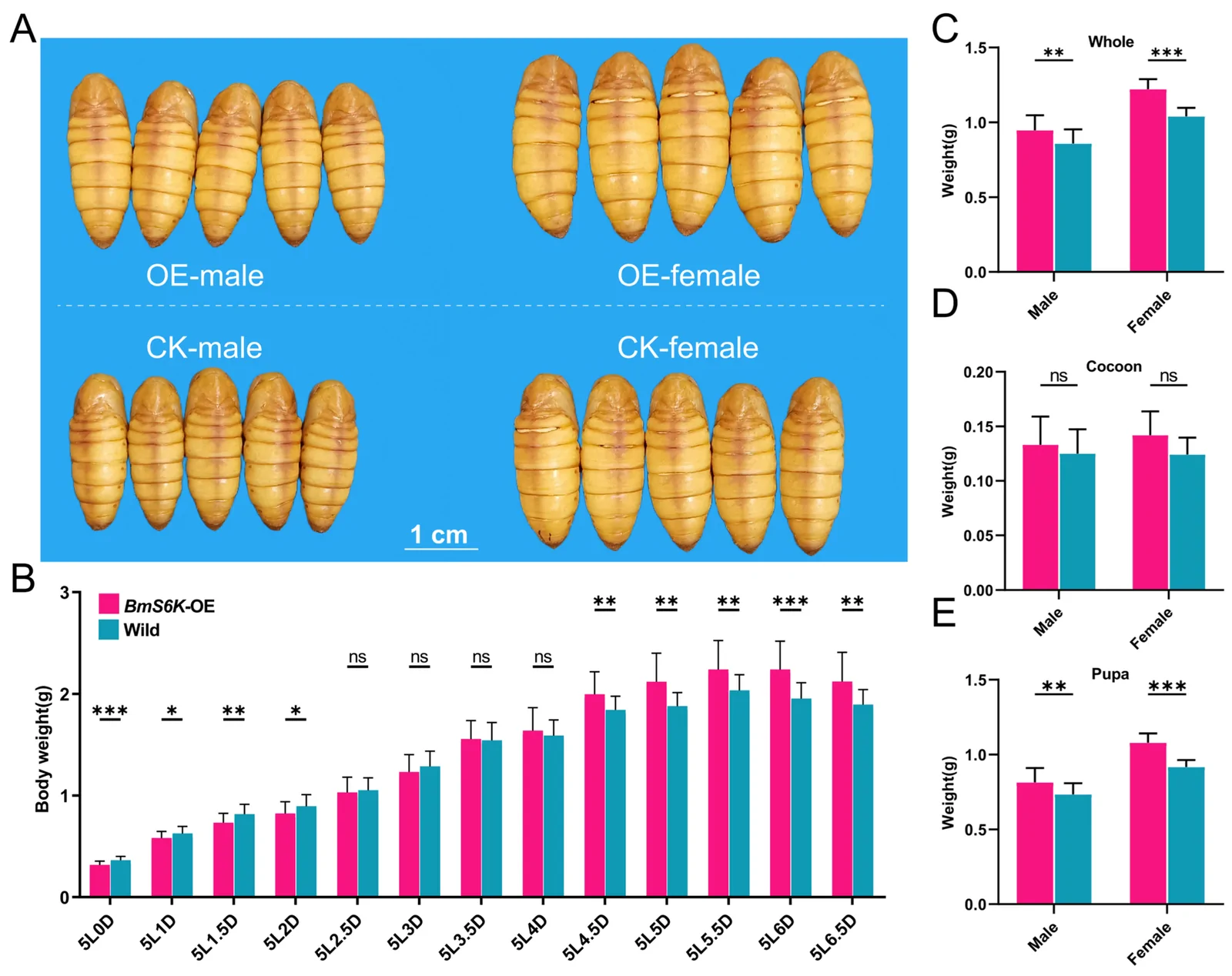
Phenotypic analysis of larval growth and cocoon–pupal traits of S6K overexpression line. (**A**) Representative morphological phenotypes of pupae from the overexpression strain and the control strain. (**B**) Body weight dynamics of fifth-instar larvae in the overexpression strain and the control strain, measured from day 0 of the fifth larval instar (5L0D) to 7 days post-wandering. (**C**–**E**) Comparisons of total cocoon weight (**C**), cocoon shell weight (**D**), and pupal weight (**E**) between male and female individuals of the two strains. ns, not significant; * *p* < 0.05; ** *p* < 0.01; *** *p* < 0.001.

Following the successful construction and molecular validation of the *BmS6K* overexpression strain, we examined its phenotypic performance at the pupal stage. Both male and female pupae of the overexpression strain were visibly larger than those of the control strain (Figure 8A). To elucidate the developmental processes underlying this phenotypic difference, we continuously monitored larval body weight from day 0 of the fifth instar onward. Transgenic larvae exhibited significantly lower body weight than control individuals at the early fifth instar. This weight disparity gradually narrowed with larval development and lost statistical significance by day 3 of the fifth instar. From the mid-fifth instar onward, the overexpression strain entered a phase of accelerated growth, in which its body weight became significantly higher than that of the control strain by the late fifth instar (Figure 8B). The average growth rate from day 0 to day 6.5 of the fifth instar increased by 16.9% compared with the controls (0.36–1.90 g vs. 0.32–2.12 g). This growth pattern confirmed that overexpression of *BmS6K* elevates the average growth rate during the fifth larval instar and drives compensatory catch-up growth. We quantified several cocoon and pupal traits to evaluate the economic impact of *BmS6K* overexpression. In both males and females, the overexpression strain showed significantly increased total cocoon weight and pupal weight compared with the Dazao control strain. In contrast, cocoon shell weight did not differ significantly between the two strains (Figure 8C–E).

## 4. Discussion

### 4.1. BmS6K Regulates Growth and Survival in a Dose-Dependent Manner in Silkworms

The present study provides robust bidirectional genetic evidence that *BmS6K* functions as a core positive regulator of growth and survival traits in silkworms. Previous work only inferred the potential roles of *BmS6K* in autophagy and viral responses from transcriptomic data [21,22], without direct functional validation at the organismal level. Here, loss of *BmS6K* caused severe growth delays, with pupal weight being reduced by 26.5% in males and 28.6% in females. Survival was also markedly impaired: compared with wild-type controls, final survival rates decreased by 44.2% in male mutants and 28.5% in female mutants. Reintroduction of *BmS6K* via phenotypic rescue restored the observed defective phenotypes, establishing a direct causal link between *BmS6K* deficiency and the observed traits.

Conversely, transgenic overexpression of *BmS6K* in the wild-type background increased the larval growth rate by 16.9% and increased several key indicators, including whole cocoon weight and pupal weight. The congruent phenotypic gradient across loss-of-function, wild-type, and gain-of-function genotypes confirms the positive regulatory role of *BmS6K* in silkworm growth and hence fitness, highlighting its tangible potential for application in sericultural activities.

### 4.2. Spatiotemporal Expression Patterns Support Tissue- and Stage-Specific Regulatory Functions

The spatiotemporal expression profile of *BmS6K* provided correlative insights into its physiological roles and was broadly consistent with the phenotypic outcomes following gene perturbation (Figure 1A). Transcript abundance in digestive and metabolic tissues (midgut, fat body, and head) gradually increased with larval development, reaching moderate-to-high levels from the mid-fifth instar to the wandering stage. At the same time, overall expression remained low in the embryonic and pupal stages. Notably, the global transcription peak across all samples occurred in the posterior silk gland at the prepupal stage, whereas silk glands showed only moderate expression during the fifth instar. This distribution pattern was consistent with the phenotypic outcomes observed following *BmS6K* perturbation.

The high *BmS6K* expression in the fifth-instar midgut supports a potential role in promoting larval growth via nutrient sensing and metabolic modulation. Moderate fat body expression aligns with its proposed association with basal anabolism and protein turnover. The marked upregulation of *BmS6K* in the prepupal posterior silk gland suggests a possible involvement in silk gland physiology; however, direct evidence for *BmS6K*-driven silk protein synthesis is absent, as cocoon shell weight was not significantly increased in the overexpression lines. Targeted follow-up studies are needed, including quantification of fibroin and sericin, measurement of silk gland size and cocoon shell ratio, and characterization of the mechanical properties of silk fibers.

The high-expression window in growth-related tissues during the mid-to-late fifth instar corresponds to the restricted growth acceleration period observed in the overexpression lines, implying potential coordination with endocrine signals (e.g., ecdysteroids, juvenile hormone) that regulate the transitions between larval stages. This tissue- and stage-specific pattern supports *BmS6K* as a candidate for targeted manipulation, with intervention confined to this physiologically sensitive window minimizing systemic disturbances.

### 4.3. Evolutionary Conservation and Lepidopteran-Specific Functional Divergence

Phylogenetic analysis, domain architecture comparison, and genomic synteny examination have consistently demonstrated the evolutionary conservation of S6K across *Lepidoptera*. *BmS6K* clusters with homologs from other lepidopteran species to form a well-supported lineage-specific clade. Its core functional domains (the kinase domain and hydrophobic motif) show a highly conserved structural organization, and flanking gene synteny is also retained across species, supporting an ancestral, conserved role of S6K in fundamental growth regulation in *Lepidoptera*.

Nevertheless, the phenotypic consequences of S6K disruption diverge substantially among taxa: whereas *Drosophila* S6k mutants are viable with reduced body size, *BmS6K* loss severely compromises survival in silkworms and impairs cocoon formation. This cocoon-related phenotype may reflect the specialized silk-producing physiological background of the silkworm. Since genetic redundancy, developmental timing differences, and strain background effects fail to account for such functional divergence of a deeply conserved TOR pathway effector, long-term domestication stands as the leading hypothesis for this phenotypic disparity. Formal validation will require population genetic analyses and comparative studies between wild and domesticated silkworm strains.

The combination of a conserved core growth function and potential lineage-specific phenotypic specialization highlights the potential utility of *BmS6K* as a target for both sericultural improvement and lepidopteran pest management.

### 4.4. Transcriptomics and WGCNA Delineate a Multi-Tissue Regulatory Network Centered on Energy Metabolism

Multi-tissue transcriptome profiling indicated that *BmS6K* modulates gene expression in a strongly tissue-specific manner. Compared with the wild-type controls, *BmS6K* knockout resulted in 136 differentially expressed genes (DEGs) in the fat body, 1028 in the midgut, and 1047 in the head. Downregulated genes outnumbered upregulated genes in all three tissues, supporting a predominant role of *BmS6K* as a positive transcriptional regulator of core functional pathways.

Functional annotation showed that the regulatory priorities of *BmS6K* were tissue-specific and were associated with physiological functions. In the fat body, *BmS6K* regulated genes involved in de novo lipogenesis, amino acid biosynthesis, proteolytic turnover, and the initiation of translation, processes closely linked to metabolic homeostasis and anabolism. In the midgut, the DEGs were enriched in detoxification, innate immunity, and ribosomal protein synthesis, consistent with the digestive and defensive roles of this tissue. In the head, *BmS6K* regulated genes related to glycosylation, lipid binding, and translational machinery. Notably, ribosomal protein S25 and fibrinogen-like protein were consistently downregulated by *BmS6K* knockout in both the midgut and head, representing conserved downstream effectors linking *BmS6K* signaling to translational efficiency and the immune response.

WGCNA identified the MEred module as the core downstream network of *BmS6K*. This module showed the strongest negative correlation with knockout status (*r* = −0.96, *p* < 0.001) and was not significantly associated with tissue type, suggesting that it acts as a universal effector module across tissues. Genes in the MEred module were significantly enriched in core energy metabolism processes, including V-type ATPase assembly, oxidative phosphorylation, transmembrane transport, and lysosomal function. The results suggest that *BmS6K* does not regulate individual genes independently, but systematically supports organismal growth and stress resilience by orchestrating a cross-tissue regulatory axis centered on energy supply, substance transport, and protein biosynthesis. These downstream functional categories are highly consistent with the canonical roles of the mTORC1-S6K signaling axis, providing in vivo evidence for the conserved function of *BmS6K* as a core downstream effector of mTORC1 in *Lepidoptera*.

### 4.5. Translational Implications and Future Directions

Our findings suggest two distinct translational strategies with clear applied value. The first focuses on genetic improvement for sericultural activities. Moderate upregulation of *BmS6K* via tissue-specific promoters or CRISPR activation, particularly targeting the midgut and silk gland during the mid-to-late fifth instar, is expected to stably increase pupal weight and support the comprehensive utilization of silkworm pupae without prolonging larval development or impairing the mechanical properties of the resulting silk. The well-defined stage-specific effect window enables precise temporal manipulation to maximize production while minimizing physiological interference. Further validation in commercial silkworm strains is required to confirm the stability of growth-promoting effects across different genetic backgrounds and rearing conditions.

The second strategy targets lepidopteran pest management. S6K homologs are highly conserved in major lepidopteran pests such as *Helicoverpa armigera* and *Plutella xylostella*. Development of dsRNA or small-molecule inhibitors against these homologous loci could achieve precise suppression of pest growth and survival. Given the conservation of S6K across taxa, future pest control applications should prioritize Lepidoptera-specific sequence regions to reduce the potential impacts on non-target organisms. In addition, well-characterized mammalian S6K inhibitors such as rapamycin derivatives can provide a mature reference for compound screening and safety assessment.

Further investigation into upstream regulatory inputs, including mTORC1 and ERK7 signaling, and identification of direct phosphorylation substrates of *BmS6K* will deepen our mechanistic understanding of this regulatory pathway and expand its applications in both sericultural activities and pest control.

## 5. Conclusions

Our study provides bidirectional genetic evidence supporting *BmS6K* as a positive regulator of growth and survival in *Bombyx mori*. CRISPR/Cas9-mediated knockout of *BmS6K* led to significant reductions in pupal weight and survival rate, and targeted reintroduction of *BmS6K* rescued the observed defective phenotypes, verifying the causal relationship between *BmS6K* deficiency and the physiological impairments. Overexpression of *BmS6K* in a wild-type background selectively accelerated larval growth during the late fifth instar and increased whole cocoon weight and pupal weight, mainly through increased pupal biomass. Importantly, cocoon shell weight remained unchanged in *BmS6K* overexpression lines. Transcriptomic analysis showed that the loss of *BmS6K* was associated with global transcriptional alterations in processes including energy metabolism, substance transport, lysosomal function, and protein synthesis. Collectively, the findings establish a functional framework for the growth-regulatory role of *BmS6K* in silkworms and provide a foundation for further mechanistic dissection and the comprehensive utilization of silkworm pupae.

## Data Availability

The transcriptomic data and corresponding sample information related to this study have been deposited in the National Center for Biotechnology Information (NCBI) database under the accession number **PRJNA1526990**.

## Supplementary Materials

The following supporting information can be downloaded at: https://www.mdpi.com/article/10.3390/biology15211953/s1, Figure S1: Construction of the *BmS6K* overexpression plasmid*BmS6K*; Figure S2: Schematic diagram for the construction of the *BmS6K* knockout, overexpression, and transgenic rescue silkworm lines*BmS6K*; Figure S3: Determination of the optimal soft-thresholding power for WGCNA; Table S1: Primer sequences; Table S2: wwPCR process and reagents; Table S3: Statistics of transcriptome sequencing QC and mapping; Table S4: Hub gene information of the red module.

## Abbreviations

**BmNPV**	*Bombyx mori* nucleopolyhedrovirus
***BmS6K***	*Bombyx mori* ribosomal protein S6 kinase
**Cas9**	CRISPR-associated protein 9
**CRISPR**	clustered regularly interspaced short palindromic repeats
**EGFP**	enhanced green fluorescent protein
**GS**	gene significance
**indel**	insertion–deletion
**IE1**	Immediate Early 1 promoter
**KME**	module eigengene connectivity
**KO**	knockout
**MAD**	median absolute deviation
**pBac**	*piggyBac* transposon
**SEM**	standard error of the mean
**sgRNA**	single-guide RNA
**SV40**	Simian Virus 40 polyadenylation signal
**TOM**	topological overlap matrix
**TPM**	transcripts per million
**VST**	variance stabilizing transformation
**WGCNA**	weighted gene co-expression network analysis
**WT**	wild-type
**5L3D**	day 3 of the fifth instar
