# Supplementary material for: BmS6K Regulates Growth and Survival Traits of Silkworm, Bombyx mori
Source: Biology (Basel). 2026 Oct 30;15(21):1953. doi: 10.3390/biology15211953 (PMC13762099; doi:10.3390/biology15211953)

Supplementary Tables

Table S1. Primer sequences

Name	Sequences (5' →3')	Steps
*BmS6K*_cds_F[a]	ggatccATGGCAGGTGTTTTTGATTT	*BmS6K* CDS recombination
*BmS6K*_cds_R[a]	gcggccgcTCATTTCAATTTCGAATATT	
1180_IEone_F[a]	atgatcatcatgacaATCAATGTCTTTGTGATGCGCG	Construction of *BmS6K* overexpression plasmid vector
1180_Sv40_R[a]	cactgaacattgtcaTAAGATACATTGATGAGTTTGG	
S6L-GSP1	TCATTTTGACTCACGCGGTCGT	Identification of plasmid vector insertion site
S6L-GSP2	CGCATTGACAAGCACGCCTC	
S6L-GSP3	AAGCGGCGACTGAGATGTCC	
UN-WWP1	CGTCTCCAGTCTCCATGTGTTCGTC	
UN-WWP2	CGTCTCCAGTCTTAGGCACAGTGTC	
UN-WWP3	CGTCTCCAGTCTAGTCAGTCAGGTC	
*BmS6K*_grna1[b]	taatacgactcactatagggCCAAGACGTTTACATTGACCgttttagagctagaaatag	*BmS6K* knockout grna
*BmS6K*_grna2[b]	taatacgactcactatagggTTCAAGTTCGTAAAATCACAgttttagagctagaaatag	
CRISPR_sgR(universal)[b]	aaaagcaccgactcggtgccactttttcaagttgataacggactagccttattttaacttgctatttctagctctaaaac	
*BmS6K*_c_F1	TCTGAAAGGTGGACTATGAACCA	*BmS6K* knockout checking
*BmS6K*_c_R1	TTCAGCCTTGGTATGTGCAGT	
*BmS6K*_qPCR_F	GAGGTGGACTATGAACCAGAAT	qPCR
*BmS6K*_qPCR_R	AGACGTTTACATTGACCTGGAT	
Ref_sw22934_F	TTCGTACTGGCTCTTCTCGT	
Ref_sw22934_R	CAAAGTTGATAGCAATTCCCT	

a.Lowercase letters indicate homologous arm.

b.Lowercase letters are universal sequences.

Table S2. wwPCR process and reagents

Type	Temperature (℃)	Duration	Cycle	Reagents
Normal PCR	98	2 min	1	
	98	10 s		
	60	15 s	35	TSINGKE TSE030 1.1×
	72	20 s		T3 Super PCR Mix
	72	5 min	1	
	4	∞	1	
Grna PCR	98	2 min	1	
Grna+CRISPR_sgR	98	10 s		
	55	15 s	31	PrimeSTAR® GXL Premix Fast
	68	15 s		
	68	5 min	1	
	12	∞	1	
WW-PCR P1	94	3 min	1	
S6L-GSP1+UN-WWP1	94	30 s		
	65	30 s	5	
	72	2 min		
	94	30 s	1	
	25	30 s	1	TSINGKE TSE030 1.1×
	72	2 min	1	T3 Super PCR Mix
	94	30 s		
	65	30 s	25	
	72	2 min		
	72	3 min	1	
	4	∞	1	
WW-PCR P2 & P3	94	3 min	1	
S6L-GSP2+UN-WWP2	94	30 s		
S6L-GSP3+UN-WWP3	65	30 s	5	
	72	2 min		
	94	30 s	1	
	40	30 s	1	TSINGKE TSE030 1.1×
	72	2 min	1	T3 Super PCR Mix
	94	30 s		
	65	30 s	25	
	72	2 min		
	72	3 min	1	
	4	∞	1	

Table S3. Statistics of transcriptome sequencing QC and mapping

Sample	Description	Raw reads	Clean reads	Clean rate (%)	Mapped reads	Mapping rate (%)	Unique mapped	Unique rate (%)
cF1	KO, Fat-Body, R1	142,632,334	136,825,732	95.93	135,945,803	99.36	129,004,921	94.28
cF2	KO, Fat-Body, R2	120,093,120	114,913,510	95.69	113,988,412	99.19	107,576,940	93.62
cF3	KO, Fat-Body, R3	128,128,922	122,624,472	95.7	121,397,831	99	115,375,980	94.09
cG1	KO, Mid-Gut, R1	99,061,868	94,816,664	95.71	94,287,068	99.44	78,402,799	82.69
cG2	KO, Mid-Gut, R2	118,212,334	112,815,802	95.43	112,233,776	99.48	91,936,665	81.49
cG3	KO, Mid-Gut, R3	115,197,122	110,352,812	95.79	109,656,921	99.37	89,429,853	81.04
cH1	KO, Head, R1	111,740,374	107,123,200	95.87	105,951,879	98.91	100,388,106	93.71
cH2	KO, Head, R2	132,680,938	127,492,488	96.09	125,812,262	98.68	118,976,324	93.32
cH3	KO, Head, R3	113,517,174	108,478,092	95.56	107,405,788	99.01	101,684,084	93.74
nF1	Wild, Fat-Body, R1	110,660,204	105,658,110	95.48	105,184,052	99.55	99,170,728	93.86
nF2	Wild, Fat-Body, R2	73,682,636	70,502,334	95.68	70,134,925	99.48	66,088,712	93.74
nF3	Wild, Fat-Body, R3	93,502,792	89,128,700	95.32	88,687,639	99.51	82,396,192	92.45
nG1	Wild, Midgut, R1	60,905,456	58,884,436	96.68	58,575,011	99.47	49,567,683	84.18
nG2	Wild, Midgut, R2	60,659,900	57,814,776	95.31	57,525,373	99.5	49,957,250	86.41
nG3	Wild, Midgut, R3	54,731,596	52,511,264	95.94	52,272,510	99.55	43,634,560	83.1
nH1	Wild, Head, R1	99,101,796	94,795,228	95.65	94,333,907	99.51	90,208,826	95.16
nH2	Wild, Head, R2	95,588,928	91,073,358	95.28	90,686,519	99.58	86,823,230	95.33
nH3	Wild, Head, R3	42,288,458	40,291,670	95.28	40,105,259	99.54	38,481,803	95.51

Table S4. Hub gene information of the red module

Gene ID [Silkbase]	Gene ID [NCBI]	Description	TOR pathway association
KWMTBOMO11051	LOC692984	Subunit of the peripheral V1 complex of vacuolar ATPase	Key regulatory component of the TOR pathway
KWMTBOMO05713	LOC101740449	Gabat, 4-aminobutyrate aminotransferase	Metabolic/regulatory network cross-talk
KWMTBOMO04972	LOC101741654	NAD-dependent protein deacylase	Metabolic/regulatory network cross-talk
KWMTBOMO05476	LOC733112	Peroxisomal membrane protein PMP22	Weak association
KWMTBOMO14080	LOC733006	ae48, alpha-esterase 48	Weak association
KWMTBOMO15509	LOC105841774	Retrovirus-related Pol polyprotein from transposon TNT 1-94	Unknown
KWMTBOMO04800	LOC105841774	Retrovirus-related Pol polyprotein from transposon TNT 1-94	Unknown
KWMTBOMO03356	LOC105841774	Retrovirus-related Pol polyprotein from transposon TNT 1-94	Unknown
KWMTBOMO16662	LOC101738420	Uncharacterized: DNA-binding transcription factor activity	Unknown
KWMTBOMO16605	LOC101737399	Uncharacterized: FAM91, N-terminal domain	Unknown
KWMTBOMO11269	LOC101737399	Uncharacterized: FAM91, N-terminal domain	Unknown

Supplementary Figures

Figure S1 Construction of the BmS6K overexpression plasmid

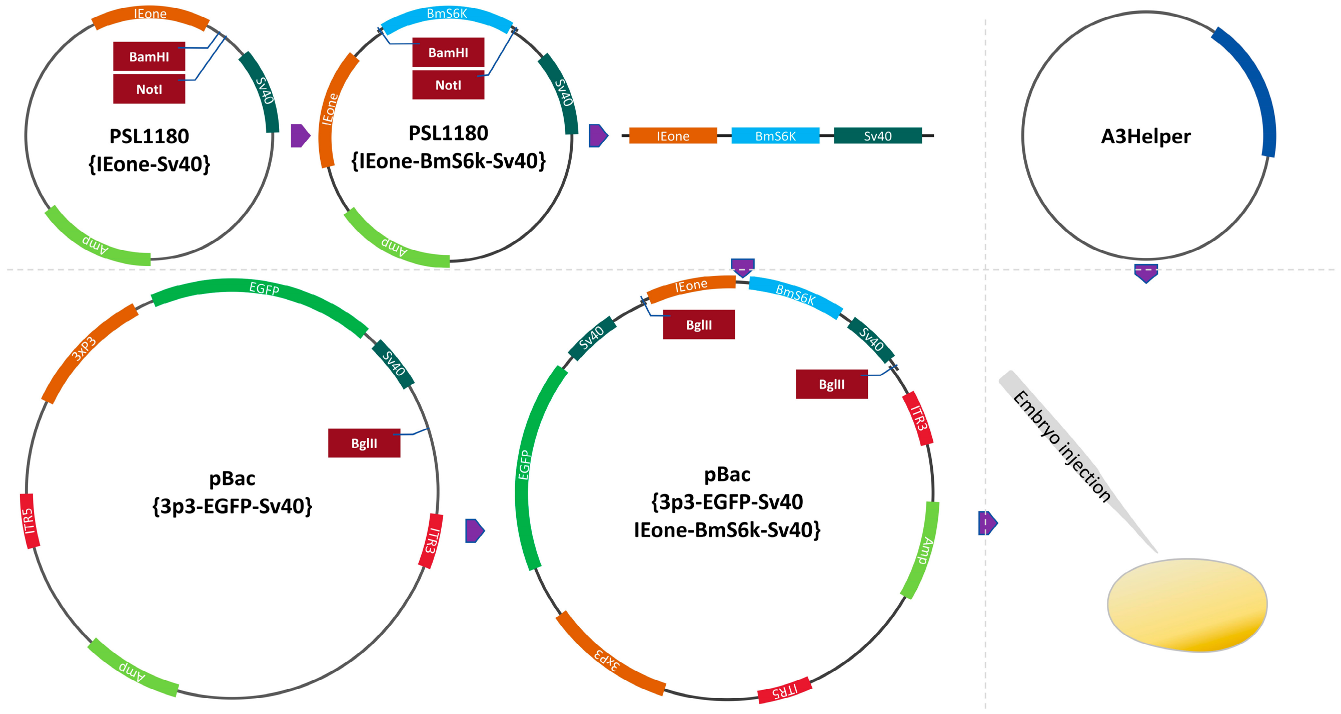

Figure S2 Schematic diagram for the construction of the BmS6K knockout, overexpression, and transgenic rescue silkworm lines.

Four experimental silkworm lines (control, knockout, overexpression, and rescue) were generated from the wild type via stepwise gene editing and genetic segregation. Chr10 and Chr22 indicate the chromosomes of the knockout and overexpression loci, respectively.

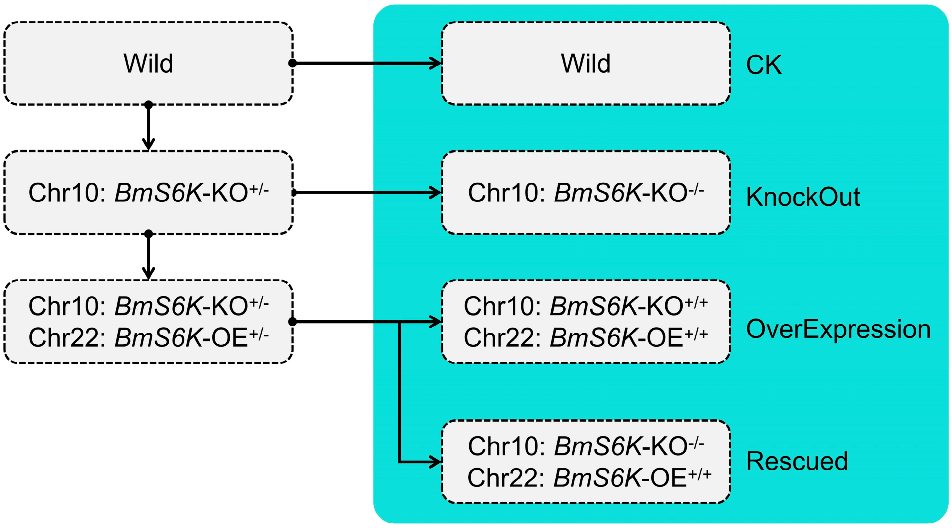

Figure S3. Determination of the optimal soft-thresholding power for WGCNA

(A) Scale-free topology model fit (signed R^2) plotted against soft threshold power (β). (B) Mean connectivity of the gene co-expression network at the corresponding soft threshold powers. A soft-thresholding power of 9 (circled) was selected to meet the scale-free network criterion for downstream co-expression module identification.

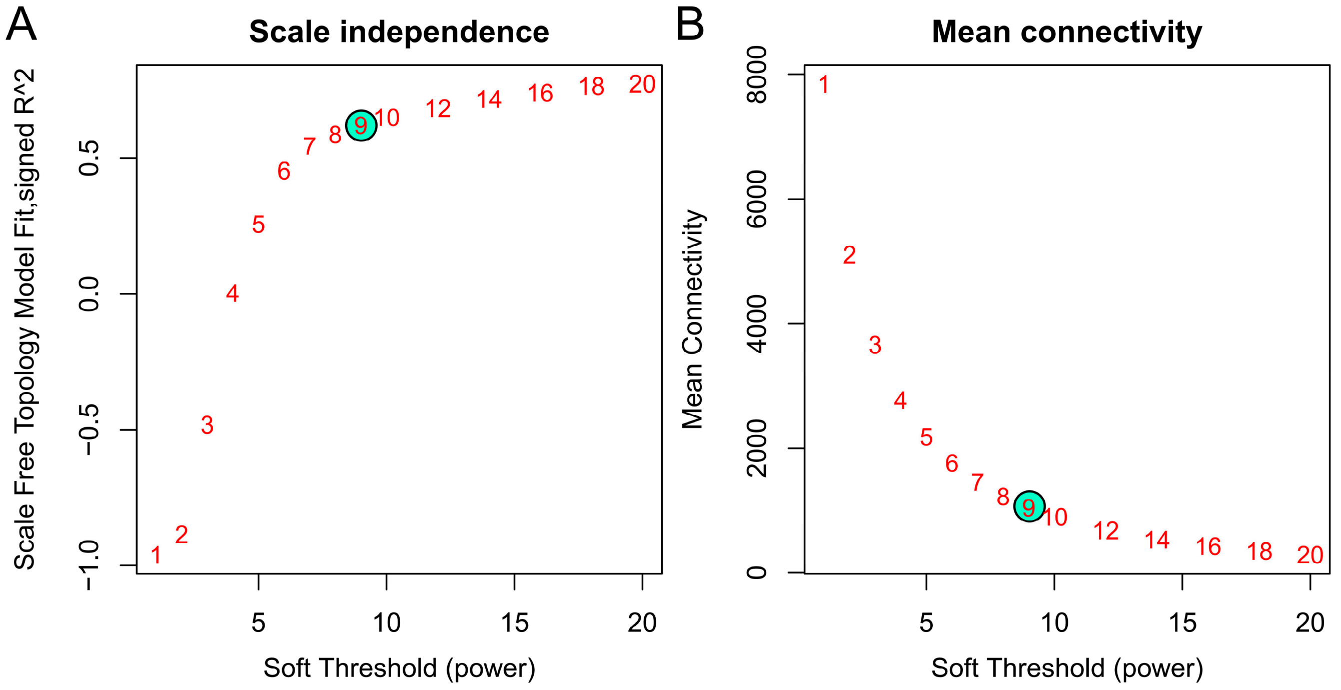

Supplement: Supplementary file 1 [file biology-15-01953-s001.zip › biology-4628542-supplementary.pdf]
